# Reduction of *Streptococcus pneumoniae* in upper respiratory tract cultures and a decreased incidence of related acute otitis media following introduction of childhood pneumococcal conjugate vaccines in a Swedish county

**DOI:** 10.1186/s12879-016-1750-5

**Published:** 2016-08-11

**Authors:** Nils Littorin, Jonas Ahl, Fabian Uddén, Fredrik Resman, Kristian Riesbeck

**Affiliations:** 1 Clinical Microbiology, Department of Translational Medicine, Lund University, Malmö, Sweden; 2 Infectious Diseases Research Unit, Department of Translational Medicine, Lund University, Malmö, Sweden

**Keywords:** Otitis media, Pneumococcal vaccines, Serogroup, *Streptococcus pneumoniae*

## Abstract

**Background:**

The effect of pneumococcal conjugate vaccines (PCV) on invasive pneumococcal disease is frequently reported, but the impact on upper respiratory tract infections in a clinical setting is less documented. Our aim in this 5-year observational study was to investigate serotype changes in a large number of *Streptococcus pneumoniae* upper respiratory tract isolates following sequential introduction of PCV7 and pneumococcal *Haemophilus influenza* protein D conjugate vaccine (PHiD-CV10) in a Swedish county.

**Methods:**

All bacterial isolates from the upper respiratory tract (nasopharynx, sinus or middle ear fluid) from patients with respiratory tract infections referred to a clinical microbiology laboratory prior to (2 years 2007–2008; *n* = 1566) and after introduction of PCV (3 years 2011–2013; *n* = 1707) were prospectively collected. Microbiological findings were compared between the two periods, and information from clinical referrals was recorded in order to explore changes in incidence of pneumococcal acute otitis media (AOM).

**Results:**

Pneumococcal serotypes covered by PHiD-CV10 decreased from 45 to 12 % prior to and after immunization (*p* < 0.001), respectively. Despite non-PHiD-CV10 serotypes increased from 49 to 80 %, a significant decline of 35 % in the absolute incidence of pneumocococal isolates (*p* < 0.001) was observed. Finally, the frequency of complicated AOM caused by *S. pneumoniae* decreased by 32 % (*p* < 0.001).

**Conclusions:**

After introduction of PCV in 2009, we have observed a significantly decreased number of pneumococcal isolates in the upper respiratory tract, a shift to non-PHiD-CV10 serotypes, and a reduction of complicated AOM. Our findings may have implications for future vaccine design.

## Background

On a global basis, *Streptococcus pneumoniae* is estimated to cause 1.6 million fatal infections annually, including 0.7–1 million children aged <5 years. The burden of disease is highest amongst children <2 years of age and in the elderly [1]. *S. pneumoniae* commonly causes invasive disease (IPD), pneumonia, septicemia and meningitis, but is also a leading cause of milder non-invasive infections such as non-bacteremic pneumonia, acute otitis media (AOM) and sinusitis. Pneumococcal infection is in most cases preceded by nasopharyngeal colonization.

Despite that more than 90 different pneumococcal serotypes have been defined, only a minority of them regularly causes severe infections. In 2001 a conjugated pneumococcal vaccine (PCV) was introduced providing protection against 7 serotypes (PCV7) and was later replaced by vaccines that target either 10 or 13 of serotypes associated with IPD, but also widespread antimicrobial resistance. PCV7 included serotypes 4, 6B, 9V, 14, 18C, 19F, and 23F, whereas the pneumococcal *Haemophilus influenzae* protein D conjugate vaccine (PHiD-CV10) includes the additional serotypes 1, 5 and 7F. Finally, PCV13 contains three more capsule polysaccharides, that is, 3, 6A and 19A.

Prevention of invasive disease in infants [2] in addition to a herd effect leading to a decreased overall incidence of IPD in the non-vaccinated population are well-documented vaccine effects [3, 4]. Moreover, studies have revealed a protective effect against pneumonia [5], sinusitis in children [6], and non-invasive vaccine type pneumonia in adults 65 years of age or older [7]. A consequence of PCV immunization programmes is, however, replacement of vaccine serotypes with non-vaccine serotypes in mainly nasopharyngeal carriage in children, but also in pneumococcal disease [8]. For example, significant increases of non-vaccine type strains causing IPD offset the reduction of PCV7 vaccine serotypes in some areas [9, 10]. Moreover, serotypes colonizing the host are known to vary geographically and temporally [11, 12], and PCV efficacy is likely different depending on baseline serotype distribution. Local surveillance of circulating strains is thus crucial to evaluate impact of PCVs.

PCV7 has been shown to reduce the incidence of AOM caused by serotypes included in the vaccine, but evidence of a decrease of all pneumococcal AOM cases irrespectively of serotype was modest in pre-clinical trials with PCV7 [13]. Importantly, a 34 % reduction in the overall incidence of AOM was found in a randomized trial with the 11-valent predecessor to PHiD-CV10 [14]. Post-marketing surveillance of the effect of widespread immunization of children with PCV7 or PHiD-CV10 have also demonstrated a substantial decrease in AOM incidence in non-institutional care and hospitals [15, 16]. However, some doubts have been brought forward questioning whether the decline in AOM can be solely attributed to PCV. Natural variations in AOM incidence, more stringent diagnostics of AOM to avoid unnecessary prescription of antibiotics, and variations in influenza epidemiology are factors that must be considered when determining the overall efficacy of PCV [17].

The aim of this study was to determine the effects on incidence of pneumococcal infections and on serotype replacement after sequential introduction of PCV7 and PHiD-CV10 in a child immunization programme. We conducted a multiyear observational study investigating the effects on respiratory tract infections in a well-defined geographical area. Changes in total pneumococcal prevalence, serotype distribution and, finally, clinical AOM were assessed.

## Methods

### Setting

In the southern county of Skåne in Sweden, immunization with the 7-valent PCV Prevenar® was initiated in January 2009 and was administered on a 2 + 1 schedule at 3, 5 and 12 months of age. It was replaced by PHiD-CV10 (Synflorix®) in June 2010, which was also administered to children who had received one or two doses of PCV7. A high coverage was obtained from the start and among children born in 2009 the coverage was 97.5 % at 2 years of age [18]. Our study was initiated in 2007 and continued through 2013. We compared 2 years before (2007/2008) with 3 years after (2011–2013) initiation of the immunization campaign.

### Data collection

All clinical isolates of *S. pneumoniae* from the upper respiratory tract (from nasopharyngeal swabs, middle ear fluid and sinuses) identified at the local Clinical microbiology lab (Malmö, Sweden) were included. One single, public clinical microbiology laboratory serves the entire geographical area. According to regional guidelines, upper respiratory tract cultures are recommended in patients with recurrent AOM, treatment failure of AOM, community-acquired pneumonia (CAP) in adults and complicated respiratory tract infections, but can also be obtained in other clinical situations at the discretion of the referring physician [19]. Routine culturing from nasopharynx in adults with suspected pneumonia admitted to hospital was included in the guidelines in 2009. Bacterial cultures obtained within a shorter interval than 2 months from the same patient were excluded.

### Study population

Pneumococcal upper respiratory tract isolates (*n* = 3273) were obtained from patients in 9 municipalities of the southwestern Skåne university hospital district. This catchment area was unchanged during the study years, while the population increased from 473,245 to 526,092 inhabitants. Sixty-seven percent were referred from outpatient clinics and 33 % from hospital infirmaries.

### Microbiology

Nasopharyngeal swabs and secretions from sinus and middle ear were transported to the Clinical microbiology lab in Amies transport medium with charcoal for culturing within 24 h on CNA agar with sheep blood medium. Pneumococcal isolates were prospectively stored at −80 °C during the study period. A minority of the isolates (21 %) was not saved and thus unavailable for serotyping. The numbers of these missing strains were more or less constant during the study years and missing isolates were considered as missing at random (range 19.9-22 %). Bacterial isolates were identified by standard methods as previously described [20]. Pneumococci were serotyped using a latex agglutination test and the Neufeld test according to the manufacturer’s instructions (Statens Seruminstitut, Copenhagen, Denmark) [21]. Both are capsular reaction tests utilizing anti-pneumococcal rabbit sera directed against the polysaccharide capsule, and are considered as the “gold standard” for serotyping of pneumococci [22]. If a negative result was obtained twice the isolate was designated as “non-typeable”. No further serotyping was performed if an isolate was fallen in the non-vaccine serogroups 10 and 35.

### Assessment of clinical diagnosis

Patients with a positive pneumococcal culture from the middle ear fluid, or from the nasopharynx in addition to concurrent typical symptoms of AOM according to the description of the referring physicians were identified. According to current guidelines, which do not recommend culturing on uncomplicated AOM, the identified AOMs in our material were in most cases complicated.

### Data analysis

Descriptive statistics, such as measures of location and spread was calculated for all variables. We pooled the pneumococcal upper respiratory tract isolates for the 2 years prior to (2007/2008) and 3 years after vaccination (2011–2013), respectively, for comparisons of absolute and relative incidences. The change in incidence rates (*p*-values), including 95 % confidence intervals (CI), after introduction of the vaccine was calculated using an exact Poisson-test performed in R version 3.2.1. To assess changes in relative frequencies of PHiD-CV10 vaccine and non-vaccine serotypes calculations were made with Fischer’s exact test using IBM SPSS Statistics 21®.

## Results

### A declining burden of pneumococcal disease

**Fig. 1:**
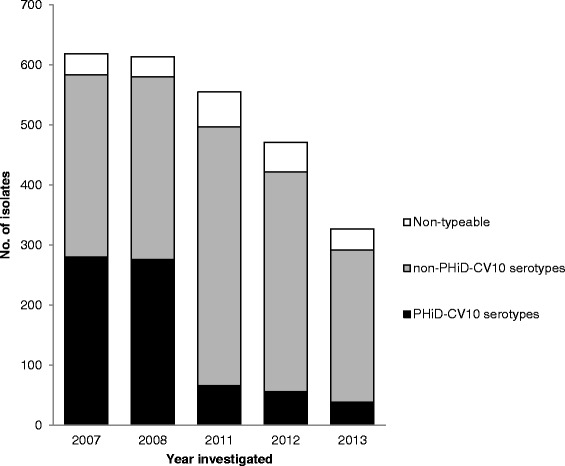
A decreased number of *S. pneumoniae* was observed during the study period. The total number of referrals for bacterial cultures (both positive and negative ones) were per year 5886 and 5965 in 2007 and 2008, respectively. During the 3 years 2011–2013 the total numbers of bacterial cultures were 5908, 5585 and 5647, respectively

An equal distribution between male and female patients (49.8 vs. 50.2 %) was observed. The median patient age at the time of sampling was 3.1 years compared to 4.2 years prior to and after introduction, respectively, of PCV in the child immunization program. Isolates from children 2 years or younger became less common between the observed time periods (Table 1), possibly suggesting a protective effect in the vaccinated population. The incidence of pneumococcal isolates decreased 35 % (*p* < 0.001) from 165 cases per 100,000 inhabitants (95 % CI: 157–173) pre-PCV to 110 (95 % CI: 104–115) post-introduction of PCV (Table 1). The annual frequency is depicted in Fig. 1 and illustrates a continued decline of pneumococcal isolates from 2011 to 2013. Importantly, the total number of culture referrals did not change during the observation period, which suggests that the number of complicated respiratory tract infections (the main indication for culturing) remained at a constant level but that *S. pneumoniae* as aetiology declined.

**Table 1:** Pneumococcal upper respiratory tract isolates analysed in this study. Incidences from 2 years prior to start of vaccination are compared to 3 years after PCV introduction

	Prior to PCV introduction (2007/2008)		After PCV introduction (2011–2013)	
Age (years)	Isolates (total number)	Incidence per 100,000 (CI 95 %)	Isolates (total number)	Incidence per 100,000 (CI 95 %)
≤ 2	884	2561 (2392–2730)	744	1250 (1161–1341)
3–16	337	229 (205–254)	330	136 (122–151)
17–40	153	51 (43–59)	234	47 (41–52)
41–64	134	45 (37–53)	265	56 (49–62)
65-	58	34 (26–43)	134	45 (37–52)
All ages*	1566	165 (157–173)	1707	110 (104–115)

*p <0.001 for difference in incidence ratio between the two time periods investigated

In order to detect cases of AOM we analyzed in detail referral texts from samples that were positive for growth of pneumococci. The physicians ordering bacterial cultures provided information on clinical disease manifestations in 85 % of cases (*n* = 2782). A significant decrease of 32 % (*p* < 0.001) in frequency of *S. pneumoniae* as aetiology for complicated AOM was found following start of vaccination. The mean annual frequency was 217 in 2007/2008 and decreased to 147 in 2011–2013.

### Serotype replacement of pneumococci

**Fig. 2:**
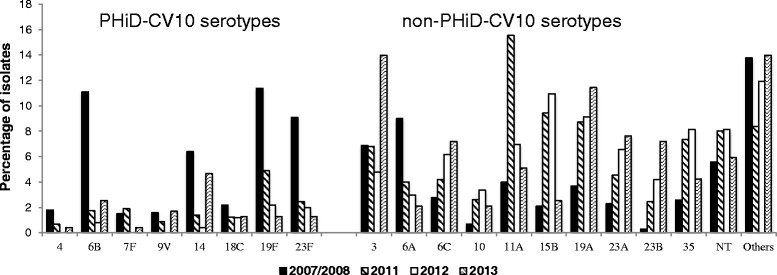
Percental distribution of *S. pneumoniae* serotypes in the present study*.* In total, 2586 pneumococci were serotyped. PHiD-CV10 serotypes 1 and 5 are not shown since they were rare (1 isolate each). Non-vaccine serotypes representing less than 2.5 % of isolates were grouped as “Others” and were, in ascending order, 2, 6D, 7C, 8, 9A, 9 N, 11B, 11C, 12, 13, 15A, 15C, 16, 17, 18A, 18B, 18F, 20, 21, 24F, 28, 31, 24, 33, 37, 38, 39, 40 and 42. NT = non-typeable pneumococci

Seventy-nine percent of pneumococcal isolates (*n* = 2586) were available for serotyping. Pneumococcal serotypes covered by PHiD-CV10 were reduced in respiratory tract infections, and to a large extent replaced by serotypes not included in the vaccine. In 2007/2008, 45 % of all pneumococcal isolates were covered by PHiD-CV10 compared to 12 % in 2011–2013 (Table 2; *p* < 0.001). In parallel, serotypes not included in PHiD-CV10 increased from 49 to 80 % (*p* < 0.001). For example, serotype 11A made up only 4 % of the serotyped pneumococci prior to introduction of PCV, but emerged as the most common serotype in 2011 (Fig. 2). Four years after PCV initiation, serotype 3 comprised 14 % of the isolates, followed by serotype 19A (11 %) and 23A (8 %). *S. pneumoniae* serotype 3 is associated with more severe disease [20], and since no significant change in the absolute numbers of serotype 3 isolates was observed when the 3 years 2011–2013 were pooled and compared to the pre-vaccine era, the proportion of serotype 3 isolates among *S. pneumoniae* increased in the post-vaccine period. The incidence of serotype 6A, which is not included in PHiD-CV10, decreased significantly (*p* < 0.001), whereas 6C was gradually more common. An increased incidence of pneumococcal serotypes and serogroups 6C, 10, 11A, 15B, 23A, 23B and 35, which are not covered by either of the currently available vaccines (PHiD-CV10 or PCV13), was also observed during the study period. Interestingly, non-vaccine type isolates increased in 2011 compared to 2007/2008 but thereafter the incidence went down in 2012 and 2013 (Fig. 1).

**Table 2:** Distribution of identified pneumococcal isolates. Two years (2007/2008) prior to PCV were compared to 3 years after (2011–2013). Of the total *S. pneumoniae* included in the present study, 78.8 % (*n* = 1234) were available for serotyping prior to and 79.2 % (*n* = 1352) after PCV introduction. *p*-values were calculated for relative incidences. Non-PHiD-CV10 serotypes that represent less than 2.5 % of serotypes before and after PCV introduction are grouped as “Others”

Serotype/Serogroup	Prior to PCV (2007/2008) % (*n*)	After PCV (2011–2013) % (*n*)	*P*-value
1	0.0 (1)	0.0 (0)	NA
4	1.8 (22)	0.4 (5)	<0.001
5	0.0 (1)	0.0 (0)	NA
6B	11.1 (137)	1.6 (21)	<0.001
7F	1.5 (19)	0.9 (12)	0.149
9V	1.6 (20)	0.7 (10)	0.043
14	6.4 (79)	1.8 (25)	<0.001
18C	2.2 (26)	1.3 (17)	0.093
19F	11.5 (141)	3.1 (42)	<0.001
23F	9.1 (112)	2.0 (28)	<0.001
Total PHiD-CV10 serotypes	45.2 (558)	11.8 (160)	<0.001
3	7.0 (86)	8.8 (119)	0.450
6A	9.1 (112)	3.3 (44)	<0.001
6C	2.9 (36)	6.7(91)	<0.001
10	0.7 (8)	2.9 (38)	<0.001
11A	4.0 (49)	10.0 (135)	<0.001
15B	2.1 (26)	9.3 (124)	<0.001
19A	3.7 (46)	9.9 (134)	<0.001
23A	3.0 (38)	6.4 (87)	<0.001
23B	0.3 (4)	4.2 (57)	<0.001
35	2.6 (32)	7.4 (100)	<0.001
Others^a^	13.8 (170)	10.9 (148)	0.111
Total non-PHiD-CV10 serotypes	49.2 (607)	79.7 (1077)	<0.001
Non-typeable	5.6 (69)	8.6 (116)	<0.001

^a^Others were in ascending order: 2, 6D, 7C, 8, 9A, 9N, 11B, 11C, 12, 13, 15A, 15C, 16, 17, 18A, 18B, 18F, 20, 21, 24F, 28, 31, 24, 33, 37, 38, 39, 40, and 42

## Discussion

We have observed changes in clinical respiratory tract infections associated with pneumococci after the introduction of PCV in a Swedish County. Bacterial cultures were referred from a well-defined catchment area served by a single microbiological laboratory, and a large number of bacterial samples were compiled during 5 years, allowing comprehensive epidemiological evaluation. Two time periods, that is, 2 years prior to and 3 years after introduction of PCV were investigated. Three major findings can be concluded; i) upper respiratory tract isolates of *S. pneumoniae* decreased by more than a third; ii) a shift in the distribution of pneumococci from serotypes included in PHiD-CV10 to non-PHiD-CV10 serotypes; and, finally, iii) that the frequency of culture-verified *S. pneumoniae* isolates in patients with symptoms of AOM decreased significantly.

The efficacy of PCV vaccines on pneumococcal syndromes such as IPD, pneumonia, AOM and sinusitis has previously been thoroughly investigated [2–7]. Our study is important since it assesses the impact of PCV on pneumococci in the upper respiratory tract of all patients with signs of respiratory infection. These patients were in our study mainly attending outpatient clinics (family physicians), where antibiotic treatment is often considered. A lower incidence of pneumococcal isolates may thus reduce the need for antibiotics.

In pre-marketing studies, evidences of any effect of PCV on AOM are scarce. Our observation of a decrease in complicated AOM due to *S. pneumoniae* is, however, in concordance with several post-licensure studies. Tamir *et al.* found a reduction in episodes of severe pneumococcal AOM [23], and others have reported a decrease in AOM-related visits to hospitals and outpatient clinics [17]. The decreased carriage of PCV serotypes in the population causes a reduced transmission of pathogenic bacteria, which could explain the decrease in AOM. It cannot be excluded that other factors, such as natural variation in AOM disease contributed to the reduction as presented in our study. Pneumococcal infection is often promoted by influenza virus. However, according to statistics from the Swedish Public Health Agency the influenza seasons were of “medium intensity” in 2007/2008 and of “high intensity” in 2011-2013 [24], and thus the observed decrease of pneumococcal isolates should not be attributed to milder influenza seasons.

The serotype replacement observed in this study is in concordance with previous publications [8]. In studies on carriage of pneumococci in the nasopharynx of children, the phenomenon of serotype replacement has been suggested to maintain overall carriage, even after introduction of PCV [25]. The vacant niche left by eradicated vaccine serotypes are replaced by non-vaccine strains. On the other hand, we analysed carriers of pneumococci having symptoms of respiratory tract infection and not passive carriage of pneumococci. The observation that non-vaccine isolates became less prevalent after introduction of PCV (Fig. 1) might be explained by a lower virulence among non-PCV serotypes as previously suggested [8]. Cross-protection of capsule polysaccharides included in the vaccine could also have contributed to the overall reduction of clinical isolates found. Previous results from studies on PCV7 and PHiD-CV10, which both contain the serotype 6B capsule, have suggested significant cross-reactivity against serotype 6A, and subsequent protection from disease caused by this particular serotype [14, 25], findings that are supported also in the present study. In contrast, pneumococci carrying the 6C capsule emerged, and it has recently been recognized as an expanding serotype in the PCV era.

Pre-marketing studies highlighted cross-protection against non-vaccine serotype 19A, which is closely related to vaccine serotype 19F. However, in some countries with high PCV7 coverage, an increased pneumococcal disease caused by serotype 19A has occurred, and questioned the existence of serotype 19F-19A cross-protection by the PCV7. On the other hand, PHiD-CV10 has been found to induce higher anti-19A IgG titers in OPA (opsonophagocytosis) compared to PCV7, suggesting a superior immune response [26]. Surprisingly, we found that the absolute and relative incidence of *S. pneumoniae* serotype19A increased during the observation period, and together with serotype 3 comprised 18.8 % of all *S. pneumoniae* 4 years after PCV introduction. Both serotypes 3 and 19A are included in PCV13, which raised the question of possible benefits for this vaccine in the study population. However, immunization with serotype 3 has been shown to induce a hyporesponse and the vaccine effect against this specific serotype is questioned [27]. Another fact is that PCVs were included in the child immunization programme in order to protect against IPD in children, whereas the focus of the present study was on upper respiratory tract isolates. In our catchment area, serotypes 11A and 15B, none of which are included in the available conjugated vaccines, were among the most common serotypes after PCV introduction, a finding consistent with previous studies which have also found them to be emerging pneumococcal serotypes [28, 29].

There are a few limitations associated with our investigation. Firstly, samples may be taken for reasons other than those recommended by regional guidelines or clinical praxis. Some physicians may also be more prone to take nasopharyngeal swabs than others. However, we believe that these confounders are unlikely to have changed between the two time periods compared. Secondly, all pneumococcal isolates were not available for typing. The percentage of lost isolates was, however, evenly distributed prior to and after the introduction of PCV, and the missing isolates were considered as missing at random.

## Conclusions

Increased incidence of non-vaccine types may affect vaccine efficacy and could have implications for future vaccine design, *i.e.,* a protein-based vaccine would be ideal with respect to this. To monitor the frequency of emerging serotypes and highly pathogenic strains such as serotype 3 is important in the post-PCV era. A related concern is a shift in disease to other bacterial species, which could potentially offset the positive effects of PCV on airway infections. This concern regarding upper respiratory tract isolates and AOM needs to be further investigated.

## Abbreviations

AOM, acute otitis media; CI, confidence interval; IPD, invasive pneumoccal disease; PCV, pneumococcal conjugated vaccine
